# Biotransformations Utilizing β-Oxidation Cycle Reactions in the Synthesis of Natural Compounds and Medicines

**DOI:** 10.3390/ijms131216514

**Published:** 2012-12-05

**Authors:** Alina Œwizdor, Anna Panek, Natalia Milecka-Tronina, Teresa Kołek

**Affiliations:** Department of Chemistry, Wroclaw University of Environmental and Life Sciences, Norwida 25, 50-375 Wroclaw, Poland; E-Mails: anna.szpineter@up.wroc.pl (A.P.); natalia.milecka@gmail.com (N.M.-T.); teresa.kolek@interia.pl (T.K.)

**Keywords:** β-oxidation, biotransformation, bioconversion, sterol side-chain degradation, steroidal pharmaceuticals, flavoring lactones, l-carnitine, β-hydroxyisobutyric acid, vanilla flavor

## Abstract

β-Oxidation cycle reactions, which are key stages in the metabolism of fatty acids in eucaryotic cells and in processes with a significant role in the degradation of acids used by microbes as a carbon source, have also found application in biotransformations. One of the major advantages of biotransformations based on the β-oxidation cycle is the possibility to transform a substrate in a series of reactions catalyzed by a number of enzymes. It allows the use of sterols as a substrate base in the production of natural steroid compounds and their analogues. This route also leads to biologically active compounds of therapeutic significance. Transformations of natural substrates via β-oxidation are the core part of the synthetic routes of natural flavors used as food additives. Stereoselectivity of the enzymes catalyzing the stages of dehydrogenation and addition of a water molecule to the double bond also finds application in the synthesis of chiral biologically active compounds, including medicines. Recent advances in genetic, metabolic engineering, methods for the enhancement of bioprocess productivity and the selectivity of target reactions are also described.

## 1. Introduction

The literature continually brings to light a growing number of cases indicating that the stereochemical structure of a given compound determines its bioactivity (among others, its olfactory properties, activity as an insect pheromone), and that chirality is an important factor in drug efficacy [1,2]. For these reasons, there is a vast range of research activity related to synthesis routes leading to the specific enantiomers of chiral biologically active compounds. Efforts within this field are directed mostly towards such major tasks as the synthesis of optically active medicines and nutrient components. The design of synthesis of a chiral drug is preceded by studies establishing the bioactivity of the stereoisomers and their metabolic fates.

The basic paradigm of synthetic schemes for optically active compounds, either those based on specially designed chiral building blocks or those employing chiral centers in naturally occurring compounds—synthesis intermediates, is stereoselectivity of the enzymatic action. Enzymes exhibit high activity as well as selectivity, including chemo-, regio-, stereo- and enantioselectivity, therefore biocatalysis is widely applied in the synthesis of optically active compounds [3]. Syntheses of food additives and substrates used in the production of natural steroid hormones and their analogs utilize reactions catalyzed by enzymes, among which the enzymes of the β-oxidation cycle are frequently found [4,5].

**Figure 1:**
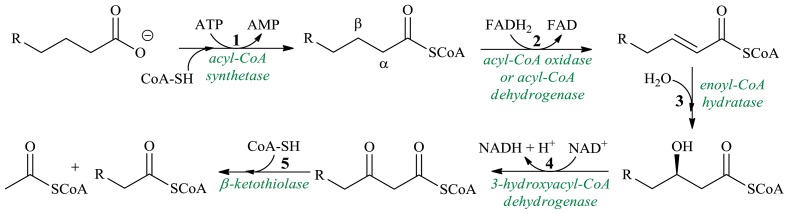
Scheme of the β-oxidation cycle.

β-Oxidation is the metabolic pathway of fatty acids oxidation by which fatty acids, in the form of acyl-CoA molecules, are broken down (Figure 1). Acetyl-CoA and a CoA thioester of an acid shorter by two carbon atoms are the products of a single round of the β-oxidation cycle of unbranched chain fatty acids. Subsequently, the shortened fatty acyl-CoA undergoes a further round of the β-oxidation cycle.

Oxidative reactions of the β-oxidation cycle are preceded by activation of the fatty acid to its thioester with coenzyme A (CoA) catalyzed by ATP-dependent ligase (Figure 1, step 1). As a result of dehydrogenation of the thioester, the enoyl-CoA is formed, and after hydration with the participation of enoyl-CoA hydratase, the hydroxyacyl-CoA is produced. The dehydrogenation reactions and the addition of a water molecule to the enoyl-CoA are stereoselective (Figure 1, steps 2 and 3). The enoyl-CoA hydratases exhibit significant enantioselectivity: the vast majority of hydratases described in the literature catalyze the addition of a water molecule to *trans* and *cis* enoyl-CoA, however the l-hydroxy product is the product of hydration of the *trans* bond, while the result of hydration of the *cis* substrate is the d-isomer. The third reaction of this pathway is the oxidation of the hydroxyl group, catalyzed by the 3-hydroxyacyl-CoA dehydrogenase. The thiolase catalyzes the thiolytic cleavage of β-ketoacyl-CoA into two molecules of acyl-CoA as products (Figure 1, step 5). The β-Oxidation process occurs in both mitochondria and peroxisomes. Generally, both models differ in metabolic fluxes. Mitochondrial β-oxidation is very efficient, usually converting R-CoA to the final product—acetyl-CoA. This pathway constitutes the major process by which fatty acids are oxidized to generate energy. Peroxisomal β-oxidation does not proceed via channelization, and its intermediates may accumulate in cells.

Xenobiotic molecules, such as certain drugs and environmental pollutants, can also be metabolized along with the fatty acids by β-oxidation in mammalian organisms. *In vitro* and *in vivo* investigations have shown that lovastatin is metabolized by rat and mouse liver microsomes to the reaction products of the β-oxidation cycle [6]. Other cholesterol-lowering drugs such as simvastatin, pravestatin, and fluvastatin are believed to undergo a typical β-oxidation of the heptanoic side chain [7]. 4-Heptanone, identified in human urine, is probably a product of the β-oxidation of 2-ethylhexanoic acid from plasticisers [8]. Last year the results of a study were published which indicate the contributions of the peroxime and β-oxidation cycle to biotin synthesis in *Aspergillus nidulans*; it is believed that an analogous synthesis also occurs in other fungal strains [9]. Many strains of bacteria metabolize carboxylic acids of various structures within the β-oxidation cycle, as well as other substrates after their conversion to the product with a carboxylic group [10,11]. These strains can use the metabolized compounds as the sole carbon and energy sources. This review mainly focuses on the application of the reaction of the β-oxidation cycle in biotransformation processes, therefore some cases are not covered, e.g., the industrially most important example is the synthesis of polyhydroxyalkanoates (biodegradable polymers) and chiral 3-hydroxyacids.

One of the major advantages of the synthetic use of β-oxidation cycle reactions is the possibility of substrate transformation along a series of reactions, catalyzed by a series of enzymes, in one biotechnological step utilizing whole cells of microorganisms. On the other hand, these biotransformation processes suffer from the fact that the desired product is further transformed in the culture of the microorganism. Typically, the desired metabolite is a reaction product of some fragment of the metabolic pathway, while the strain produces enzymes catalyzing the whole path of substrate oxidation to CO_2_ and H_2_O. Further degradation of the freshly formed product, formation and accumulation of side products, inhibition of enzymatic activity at higher concentrations of the product and its toxicity are the major factors limiting efficiency of such biotransformation processes. Research is aimed at development of the operating conditions for production of a selected valuable product with the highest performance (e.g., to determine the effect of pH and dissolved oxygen concentration, which is necessary for the regeneration of the cofactors FAD and, more indirectly, NAD^+^). Mutants are selected, or recombinant biocatalysts are constructed, which exhibit a deficit of enzymes catalyzing further transformation of the desired product or synthesis of side products. Process workflow methodologies are also developed to reduce the toxicity of the product towards the biocatalyst.

## 2. Production of Optically Active Compounds

### 2.1. Syntheses Employing Chiral Stereogenic Centers of a Natural Product

The majority of chiral natural compounds are present in only one specific enantiomeric form, as they are products of enzymatic reactions. Chiral center(s) present in a molecule of a natural optically active compound can be a base for a number of syntheses of optically active compounds. For example, d-glucose is a substrate in the syntheses of, among others, l-ascorbic acid (vitamin C) [12] and optically active 1-deoxynojirimycin [13]. The current article discusses the synthesis of flavoring lactones from lipids as well as selective degradation of the sterol side chain to C-19 or C-22 product(s). The pharmaceutical industry has expressed interest in selective sterol side chain degradation, because this process results in formation of steroid intermediates which can be used as building blocks for further synthesis of bioactive steroids of medicinal use.

#### 2.1.1. Production of Flavor-Active Lactones

Use of biocatalysts in the production of flavoring compounds structurally identical with those present in natural sources is preferred, because of, among others, formal (regulatory) reasons. According to the U.S. and European regulations (e.g., CFR 1990 and EEC 1334/2008), compounds isolated from natural resources, or obtained in microbial or enzymatic processes involving precursors isolated from nature, are classified as “natural”. Flavoring compounds found in nature, but obtained by chemical routes, are referred to as “nature-identical”. Consumer preferences reflecting the trends towards “healthy lifestyle” decide that the vast majority of fragrance food additives are compounds classified as “natural”. Lactones are a family of flavors and fragrances that are affected by such a trend. They are widely distributed in food, beverages and cosmetic preparations. Among these compounds one can find decalactone (4-decanolide), which provides a suitable characteristic aroma of peaches, apricots or strawberries. The price of γ-decalactone was 20 thousand US$/kg before the development of biotechnological methods of its synthesis. The possibility of producing this lactone from natural oils reduced the price of this product down to 1.2 thousand US$/kg (1986) and after the optimization of the fermentation, there was a further reduction of this price to 500 US$/kg (1998) and ca 300 US$/kg in 2004 [4,14].

A general metabolic pathway for the production of lactones from hydroxy fatty acids consists of the β-oxidation cycle and intramolecular esterification. Depending on the position of the hydroxyl group of the carboxylic acid, lactonisation can give γ-, δ- or ɛ-lactones. It is worth mentioning that lactones obtained by biocatalysis have a very high optical purity—their stereochemistry is determined by chirality of the asymmetric centers of their hydroxy fatty acid precursors.

**Figure 2:**
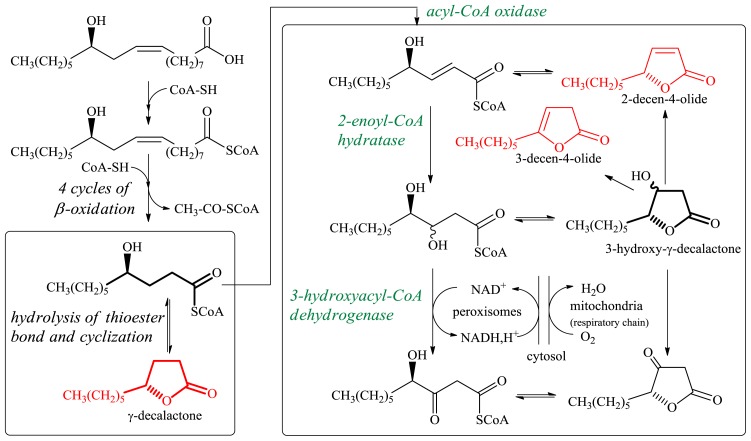
Biochemical pathway of the formation of different lactones from ricinoleic acid (modified from [20]). The reactions and enzymes implicated in β-oxidation at the C_10_ stage are shown in the rectangles.

Studies on the development of the efficient synthesis of γ-decalactone by biocatalysis were started by selecting a strain capable of conversion of ricinoleic acid (or its methyl ester) to 4-hydroxydecanoic acid [15], which can be lactonised spontaneously under acidic conditions (pH 2) and/or after heating. Ricinoleic acid is one of only a few natural hydroxy fatty acids available in large quantities. The hydrolysis product of castor oil contains about 90% of this acid. The transformation of ricinoleic acid to 4-hydroxydecanoic acid includes four β-oxidation cycles; at each cycle a two-carbon-shorter metabolite—SCoA and an acetyl-CoA are produced (Figure 2).

Many processes using the bioconversion of ricinoleic acid to produce γ-decalactone have been patented (see in [16]). The oxidation of ricinoleic acid is conducted by microorganisms belonging to *Yarrowia*, *Saccharomyces*, *Candida*, *Rhodotorula*, *Sporobolomyces*, *Monilia*, *Pichia*, *Aspergillus*, *Mucor* and *Cladosporium* genera. The processes with the highest product concentrations use *Candida sorbophila*[17] and *Yarrowia lipolytica* strains [18,19]. The conversion of ricinoleic acid by *Candida sorbophila* can produce about 50 g/L of γ-decalactone [17]. The maximum production of γ-decalactone by *Yarrowia lipolytica*, in the optimum conditions of agitation and aeration, was more than 11 g/L in less than 70 h [19].

There are two main reasons for commonly much lower (up to 4–5 g/L) yields of γ-decalactone. The first one is the degradation of newly synthesized lactone by the producing yeast. The second one is only a partial use of ricinoleic acid or intermediate at the C_10_ level, which is simultaneously the precursor for other γ-lactones (3-hydroxy-γ-decalactone and the products of its dehydration—dec-2-en-4-olide and dec-3-en-4-olide) (Figure 2).

The β-oxidation loop in the mitochondrial catabolism of fatty acids is theoretically repeated until the complete degradation of the substrate. In yeast, peroxisomal β-oxidation does not proceed via channelization, and at each stage, the metabolite can be hydrolyzed and accumulated depending on the pool of free CoA and acetyl-CoA, the pool of necessary cofactors or the saturation of the next enzyme of the cascade [21]. Thus, the amount of produced lactone is a result of competition between different reactions. The research on the improvements in the productivity of γ-decalactone focuses on the role of the genes that encode the isozymes of acyl-CoA oxidase (Aox1 to 6, encoded by *POX1* to *6*[22]) catalysing the first step of β-oxidation. Aox is generally considered as the fundamental rate-limiting enzyme. The best-known ones are the Aox isoenzymes from *Yarrowia lipolytica*. The specific role of each acyl-CoA oxidase was presented in several reviews [14,21]. It was confirmed that short-chain-specific Aox3 is involved in the oxidation after the C_10_ level and the disruption of the *POX3* gene decreases lactone degradation [23,24]. Aox4 and Aox5 are non-chain-length-specific acyl-CoA oxidases and their activity is weak, albeit directed towards the wide range of substrates, whereas Aox1 is inactive [25]. The long-chain-specific Aox2 was significant for conversion of ricinoleic acid and hence for the production of γ-decalactone. Deleting all the *POX3–5* genes resulted in an increased accumulation and an inhibition of γ-decalactone degradation [22,26]. The designed mutant produced 10 times more lactone than the wild type, and its growth was only slightly altered in comparison to the native strain. Recently, a recombinant of the diploid strain *Y. lipolytica*, with expression of *POX2* gene and disruption of *POX3* genes on two chromosomes (but without disruption of *POX4* and *POX5* genes) was constructed, and this mutant could be grown in the continuous fermentation of methyl ricinoleate. Compared with the wild type, the production of γ-decalactone was increased 4-fold, and there was no re-consumption of the product. It could be concluded that Aox2’s positive effect had a greater influence than the Aox3’s negative action to the γ-decalactone production [27].

Another problem is the modification of β-oxidation flux, which allows a shift in the equilibrium between production of γ-decalactone and production of 3-hydroxy-γ-decalactone. It can however be achieved by decreasing the Aox2 and Aox3 activity. For a mutant with disrupted *POX2* and *POX3* genes the production of hydroxylactone was minimized [14,21,24]. It was confirmed that accumulation of 3-hydroxy-γ-decalactone occurs when the amount of oxygen is lowered [20,21]. Low aeration conditions (e.g., during cell growth) resulted in low 3-hydroxy-acyl-CoA dehydrogenase activity, because its cofactor regeneration (NAD^+^) is not sufficient (Figure 2). This cofactor is regenerated through a shuttle mechanism, which probably depends on mitochondrial respiration. Under the hypoxiation regime, a lack of oxygen stopped the fluxes of the oxidation cascade at earlier stages and causes the accumulation of γ-decalactone. 3-Hydroxy-γ-decalactone is the precursor of two decenolides with flavoring properties (Table 1). Although both decenolides are characterized by interesting sensory properties, they are not commercially produced because of the lack of simple methods for their separation [14].

Apart from β-oxidation activity, another factor influencing the yield is the toxicity of the γ-decalactone and its C_10_-precursor against the producing strains. The mechanism involved in this toxicity is linked with the fact that the side-chain of the lactone interacts with cellular membranes, increasing their fluidity and decreasing their integrity [28]*Sporidiobolus* strains are particularly sensitive to the presence of γ-decalactone [29]. A number of strategies were developed to reduce the cytotoxity of γ-decalactone (e.g., adsorption on porous hydrophobic sorbents, inclusion in β-cyclodextrins, utilization of immobilization, addition of natural gum, surfactant and natural inert oils—mainly hydrogenated coconut oil or a mixture of tripalmitin, tristearin, triolein). For more details we refer the reader to the recent reviews and research articles [14,21,27,30].

**Figure 3:**
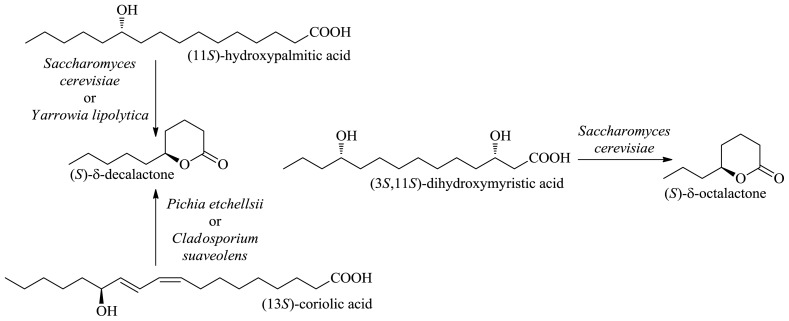
Formation of saturated δ-lactones from hydroxy fatty acids.

Besides γ-decalactone, many other lactones can be produced by biotechnological processes (Table 1). Their price depends on the availability of the corresponding hydroxy fatty acids. Moreover, microorganisms can also perform the step for the introduction of the hydroxyl group, if adequate substrates (hydroxyacids) are not available.

(11*S*)-Hydroxypalmitic acid (jalapinolic acid) and (3*S*,11*S*)-dihydroxymyristic acid (ipurolic acid) can be extracted from a jalap resin or sweet potato for the purpose of their use in producing natural (*S*)-δ-deca- and (*S*)-δ-octalactone, respectively [31]. (*R*)-δ-Decalactone can be obtained by the β-oxidative degradation of (13*R*)-coriolic acid, which is the major fatty acid of the seed oil of *Coriaria nepalensis*, whilst (*S*)-δ-decalactone can be produced from (13*S*)-coriolic acid of *Monnina emarginata* seed oil [32] (Figure 3).

**Figure 4:**
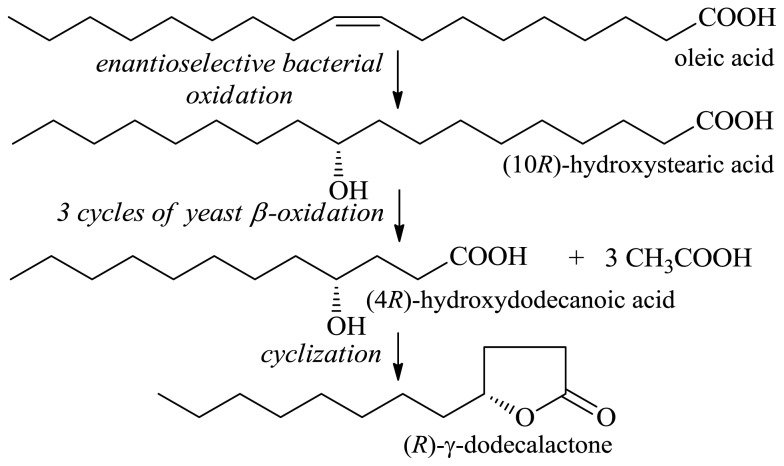
Conversion of oleic acid to (*R*)-γ-dodecalatone by biocatalysis.

Industrially useful lactones can be readily produced in large amounts from inexpensive fatty acids. γ-Dodecalactone is obtained from oleic acid in a process involving two biocatalysts [33,34]. A Gram-positive strain of bacteria catalyzed the conversion of oleic acid to (10*R*)-hydroxystearic acid, which was subsequently oxidized by bakers’ yeast to (4*R*)-hydroxydodecanoic acid (Figure 4). After cyclization, (*R*)-γ-dodecalactone was obtained in over 22% yield with respect to the initial substrate, oleic acid [33].

Many studies have focused on 10-hydroxystearic acid production from oleic acid. Recently, it has been reported that whole cells of recombinant *Escherichia coli* containing oleate hydratase from *Stenotrophomonas maltophilia* produced 49 g/L of 10-hydroxystearic acid in 4 h, with a conversion yield of 98% and a volumetric productivity of 12.3 g/L/h—the highest thus far among cells, which can make a significant contribution in the industrial synthesis of this hydroxyacid [35].

**Figure 5:**
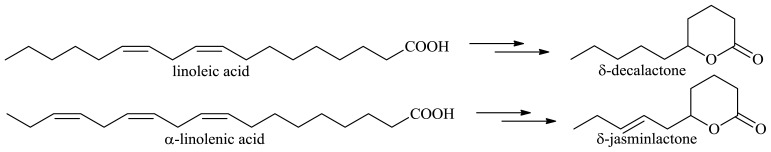
Formation of saturated δ-lactones from unsaturated fatty acids.

Linoleic acid and α-linolenic acid can be converted using microorganisms belonging to the genus *Pediococcus* or *Bifidobacterium* into 13-hydroxy-9-octadecenoic and 13-hydroxy-9,15-octadecadienoic acids, which are good substrates for the production of δ-decalactone and δ-jasminlactone by microorganisms belonging to the genus *Kluyveromyces* or *Zygosaccharomyces*, respectively [36] (Figure 5).

**Figure 6:**
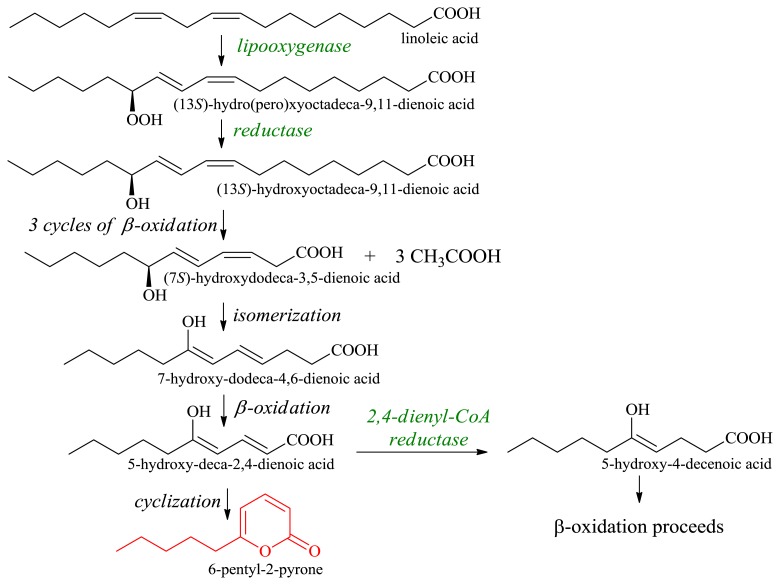
6-Pentyl-2-pyrone generation from linoleic acid by the *Trichoderma* species (adapted from [37])

Using different vegetable oils containing hydroxylated or non-hydroxylated fatty acids, it is possible to produce 6-pentyl-2-pyrone—an unsaturated δ-lactone with a strong odor of coconut [37,38] (Figure 6).

The biosynthesis of this lactone from linoleic acid in the *Trichoderma* species is initiated by a lipoxygenation at C-13 of the fatty acid with the formation of 13-hydroperoxide. The lipoxygenase reaction is followed by β-oxidation and isomerization to form 5-hydroxy-deca-2,4-dienoic acid. Low activity of the NADPH-dependent 2,4-dienyl-CoA reductase is essential for achieving good performance and yields of 6-pentyl-2-pyrone.

#### 2.1.2. Microbiological Degradation of the Side Chain of Sterols

**Figure 7:**
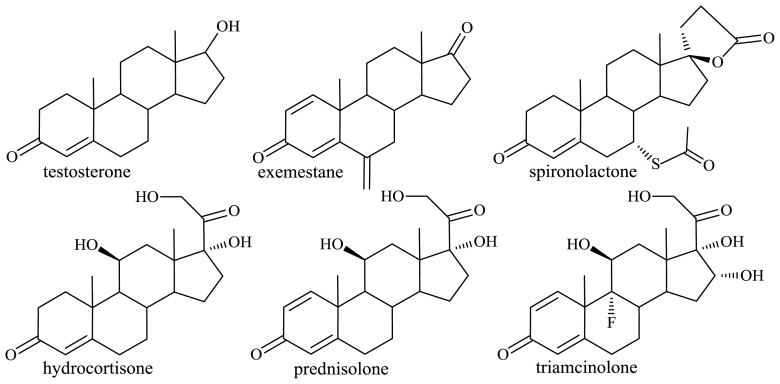
Structures of important C_19_ and C_21_ steroid hormones and their selected analogues with therapeutic action.

Steroids secreted in animals by the sexual organs and adrenal cortex (e.g., sex hormone—testosterone) play a critical regulatory role in development of reproductive structures, they also influence many aspects of metabolism and immune function (hydrocortisone—glucocorticoids), help maintain blood volume and control renal excretion of electrolytes (mineralocorticoids). Steroid compounds have a wide therapeutic activity spectrum, namely anti-inflammatory, anti-allergy, anti-fungal, anti-viral, anti-tumor, neuroprotective and immunosuppressive. They are used as agents for prevention and therapy of some disorders, for example neurodegenerative diseases, hormone-dependent breast and prostate cancer, colon cancer; metabolic, cardiovascular and central nervous system disorders (Figure 7). Steroids, frequently found among the most marketed medical products, constitute the second largest group of pharmaceuticals, next to antibiotics [39].

**Figure 8:**
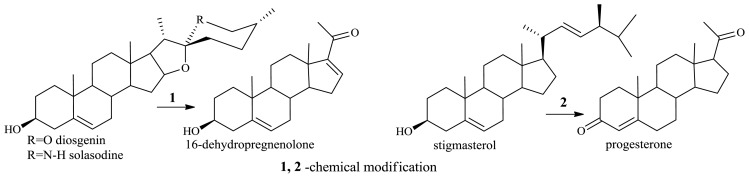
Diosgenin, solasodine and stigmasterol are chemically transformed to C_21_ products.

Availability of raw materials is one of the fundamental issues related to the production of steroid drugs. The majority of hormones and steroidal drugs are synthesized by semi-synthesis using natural steroids as starting material. Sapogenins and sterols produced in large scale by plants are the raw material in the production of steroidal drugs. Sapogenins (which include diosgenin, hecogenin, solasodine) and stigmasterol (a sterol with the double bond in the side chain) are chemically transformed to C_21_ products (16-dehydropregnenolone, progesterone), which are convenient intermediates in the synthesis of steroidal pharmaceuticals [40] (Figure 8).

**Figure 9:**
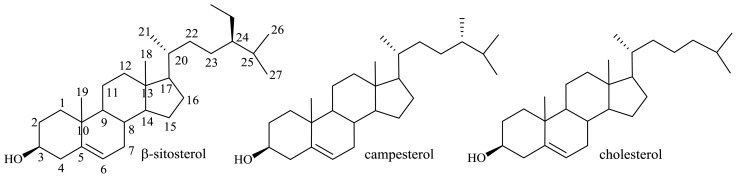
Structures of (phyto)sterols with a saturated side chain.

The limited and unstable supply of diosgenin (for many years the major raw material in steroid production), together with the increase in the production of steroidal pharmaceuticals, were impulses to carry out research on transformations of (phyto)sterols with a saturated side chain (β-sitosterol, campesterol, cholesterol) to give valuable steroidal pharmaceutical intermediates (Figure 9). Phytosterols are the major raw materials resulting from the processing of vegetable oils, from sugarcane, and from the paper industry. A deodorizate waste product of vegetable oil processing can contain over 95%, while tall-oil effluent of a paper pulp industry—over 80% of phytosterols—mainly β-sitosterol, stigmasterol, campesterol [41].

**Figure 10:**
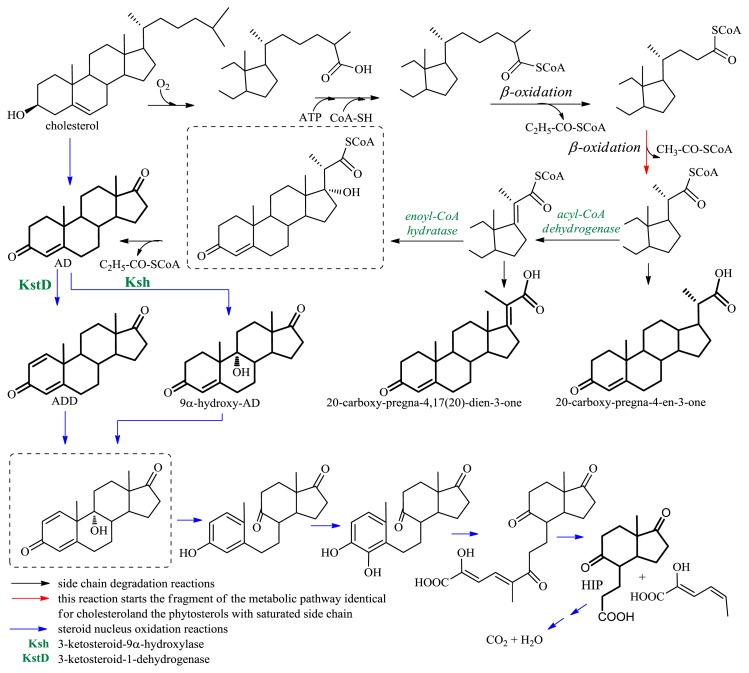
Microbial catabolic pathway of cholesterol (structures of valuable steroid drug intermediates marked by thickened line; the symbols of key enzymes in the steroid nucleus decomposition pathway are given).

In sterol molecules there is a double bond at C-5 and a hydroxyl group at the 3β-position, which can be easily converted to a 4-en-3-oxo moiety, present in the majority of steroids used in clinical settings [40] (Figure 7); unfortunately, attempts failed to develop effective methods for chemical degradation of the 17β-aliphatic side chain of sterols [42]. It was rational to assume that, due to the selectivity of enzymes, it would be possible to carry out selective degradation of the aliphatic side chain of sterols by microbial transformation. A number of strains of Gram-positive (*Rhodococcus*, *Mycobacterium*, *Streptomyces*, *Brevibacterium*) and Gram-negative (*Pseudomonas*, *Comamonas*, *Burkholderia*, *Chromobacterium*) bacteria can use sterols as the sole carbon source [43] (Figure 10). CO_2_ and H_2_O are the final products of microbial degradation of sterols, but among the metabolites there were identified synthetically useful C_19_ steroids: androsta-4-en-3,17-dione (AD), androsta-1,4-dien-3,17-dione (ADD), 9α-hydroxy-androsta-1,4-dien-3,17-dione (9α-hydroxy-ADD), or the C_22_ products: 20-carboxy-pregna-4-en-3-one, 20-carboxy-pregna-4,17(20)-dien-3-one. Also 3aα-H-4α(3′-priopionic acid)-7aβ-methylhexahydro-1,5-indanedione (HIP)—the product of partial degradation of steroidal skeleton—is the starting compound for the synthesis of medically important steroids, such as 19-norsteroids [44] (Figure 10).

**Figure 11:**
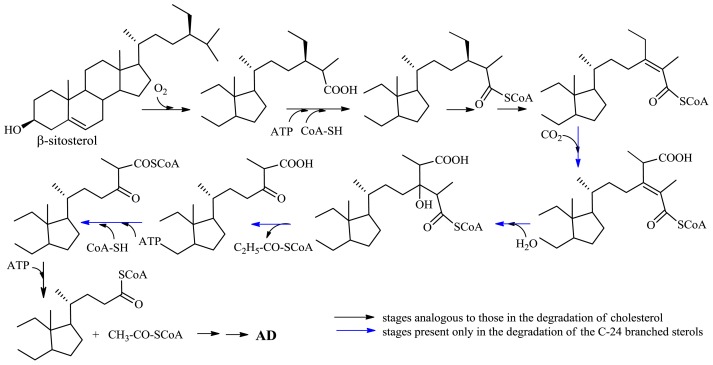
Initial fragment of the catabolic pathway of the C-24 branched sterols.

During microbiological transformation, cleavage of the aliphatic side chain of sterols occurs as a result of β-oxidation of an acid containing the carboxyl group at C-26; the said acid is formed in two subsequent redox reactions: hydroxylation at C-26 and oxidation of the introduced hydroxyl group to the carboxyl function [42]. AD is obtained as the result of oxidative degradation of the side chain of cholesterol after subsequent cleavage of propionyl-CoA, acetyl-CoA and propionyl-CoA, respectively. In the process of degradation of the C-24 branched sterols (β-sitosterol, campesterol) to AD, the C-28-carboxylation additionally occurs and there are released two molecules of propionyl-CoA, then acetyl-CoA, and finally propionyl-CoA; initial stages of degradation of the side chain of C-24 branched sterols and cholesterol are analogous (Figure 11). After formation of enoyl-CoA, before the first propionyl-CoA molecule is released, carboxylation of the allylic position (C-28) occurs. Subsequently, the product of hydration of the double bond releases propionyl-CoA in the course of a retro-aldol reaction. After CoA activation of the simultaneously formed β-keto-C26-oic product, its side-chain degradation occurs to AD, as in cholesterol [45] (Figure 11).

Many research groups experimented on selecting a strain able to carry out selective degradation of the sterol side chain to product(s) with a conserved steroid nucleus. Hundreds of microorganisms were tested, some among them efficiently metabolized sterols, but none of the studied strains converted a sterol to either a C_19_ or C_22_ steroid as a major product. Studies on microbiological degradation of sterols established that during transformations by wild-type strains, besides oxidative degradation of the side chain of the sterol, also oxidation of the steroid nucleus occurs. The key role in the process of steroid nucleus degradation is played by oxidative reactions in the rings A and B: 1,2-dehydrogenation and 9α-hydroxylation. 9α-Hydroxy-androsta-1,4-dien-3,17-dione (9α-hydroxy-ADD) is a structurally unstable chemical, spontaneously reacting by opening of ring B and aromatization of ring A (Figure 10). Inhibition of activity of 3-ketosteroid-9α-hydroxylase (Ksh) or/and 3-ketosteroid-1-dehydrogenase (KstD) is significant in the design of microbiological transformations of sterols leading to industrially valuable intermediates (C_19_ or C_22_ steroids).

During transformation of cholesterol by *Mycobacterium phlei*, in the presence of α,α-dipyridyl, 8-hydroxyquinoline or Ni^2+^ cations—9α-hydroxylase inhibitors, a mixture of AD and ADD was obtained with a good yield [11]. The accumulation of AD and ADD suggests that the side-chain degradation occurs prior to sterol ring degradation. Due to environmental concerns and production costs, optimal methods of selective degradation of the sterol side chain are such transformations in which the biocatalysts are mutants of wild-type strains with either 9α-hydroxylase or 1,2-dehydrogenase deficit (or both). After isolation of the first mutant of *Mycobacterium* sp. which performed degradation of sterols to AD, a number of mutants were described and patented [11,46]. These mutants were obtained by chemical or physicochemical mutagenesis of the native strains performing effective degradation of sterols. After identification in 2000 and 2002 of the KstD and Ksh genes responsible for the catabolism of sterols in *Rhodococcus erythropolis* SQ1 [47–49], rapid progress has been noted in the studies on identification of the genes involved in microbial degradation of sterols [50–52]. In 2007, a wealth of information on sterol catabolism was provided by the discovery of the existence of a gene cluster encoding sterol catabolism in *Rhodococcus* and *Mycobacterium* species [51]. Further, a steroid-coenzyme A ligase (FadD19) was identified and characterized as having an essential *in vivo* role in the degradation of the side chains of C-24 branched-chain sterols in *Rhodococcus rhodochrous* DSM43269 [52]. Currently, conventional mutagenesis based on random mutations induced by UV/chemical treatments is more and more frequently replaced by targeted disruption of genes encoding either Ksh or KstD to generate highly selective strains [50,53]. Genetic manipulation is potentially able to overcome a problem, significant for industrial applications, connected with the fact that most of the mutant strains in practical use produce AD and ADD simultaneously [46]. A mutant of *Mycobacterium neoaurum* NwIB-01 after inactivation of the KstD-encoding gene produced mainly AD (AD:ADD 11:1). Another mutant of *M. neoaurum* NwIB-01, in which the KstD was augmented, proved to be a good ADD-producing strain (AD:ADD 1:50); during transformation by *M. neoaurum* NwIB-01 a mixture of AD and ADD was formed with a 1:2.5 ratio [54]. An important pharmaceutical androgen—testosterone—can also be the product of microbiological transformation of sterols. Testosterone is obtained from the AD(D) either by a four-step chemical synthesis, or using microbial 17β-reduction by yeasts or fungi [55]. Microbial conversion of sterols to testosterone by using the strains capable of both sterol side chain cleavage and 17β-reduction of AD allows formation of testosterone in one biotechnological step. The strain of *Mycobacterium* sp. VKM Ac-1815D, capable of sterol side chain degradation and expressing 17β-hydroxysteroid dehydrogenase activity, converted β-sitosterol (5 g/L) to testosterone with 50%–55% molar yield [55].

**Figure 12:**
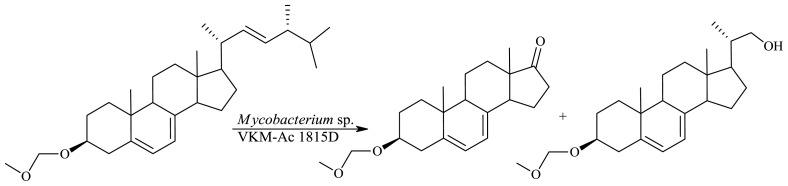
Degradation of 3β-methoxy-methoxy-ergosterol to products with the 5,7-diene system.

Ergosterol—a sterol with the 5,7-diene system and a double bond in the side chain—is produced mainly by fungi and phytoplankton. Some strains able to catalyze transformation of β-sitosterol to C_19_ products were also able to oxidize ergosterol to AD [56,57]. During conversion of ergosterol, besides side chain degradation and transformation of the 3β-hydroxy-5-ene to the 3-oxo-4-ene moiety, hydrogenation of the double bond at C-7 also occurred. Conversion of ergosterol led to much lower yields of AD when compared to the sterol substrates mentioned earlier, therefore reports on microbiological degradation of ergosterol are rare [57]. Products of transformations of ergosterol derivatives (the C_3_-OH hydrogen atom substituted by a group which is not eliminated under the conditions of the transformation) by wild-type strains can be C_22_ and/or C_19_ products with a preserved 5,7-diene system [57,58] (Figure 12).

Transformation of 3β-methoxy-methoxy-ergosterol by the strain *Mycobacterium* sp. VKM Ac-1815D led to a mixture of 3β-methoxy-methoxy-androsta-5,7-dien-17-one (the major metabolite) and (20*S*)-3β-methoxy-methoxy-20-hydroxymethyl-pregna-5,7-diene [57]. Modification of the ring A structure of ergosterol has not exerted significant influence on the process of side chain degradation by *Mycobacterium* sp. VKM Ac-1815D; the major product was obtained with 60% yield, at the 5 g/L substrate concentration. 3β-Hydroxy-androsta-5,7-diene-17-one is a key intermediate for the synthesis of novel vitamin D derivatives. Naturally occurring forms of vitamin D (*i.e.*, 1α,25-dihydroxylated ergo- or cholecalciferol derivatives) can produce hypercalcemia or phosphatemia when administered in pharmacologically relevant doses. For this reason, natural and synthetic analogs of vitamins D_2_ and D_3_, called deltanoids, have been actively investigated, and modifications include the rings as well as the side chain of the vitamin [59,60].

Since the 1980s, products of microbiological degradation of sterols have found practical applications [5]; currently, almost half of the starting materials for steroid drug production are obtained by the bioconversion process of sterols [40]. Efforts are continuing to increase the efficiency of the process of microbiological degradation of sterols to valuable steroids. The efficiency of the enzymatic transformation of sterols is limited mainly by the low water solubility of (phyto)sterols and the fact that the final products are toxic to microorganisms. The preferred method of enhancing the bioavailability of phytosterols by improving their solubility is surfactant-facilitated emulsification [61,62]. During the transformation process, sterol uptake occurs by direct contact between microbial cells and the phytosterol particles [61,63,64]. Therefore some microorganisms can use poorly water-soluble hydrocarbons as carbon sources, especially if these microorganisms have the following features: lipophilic cell walls, active transporters and membrane-associated enzymes, as well as the capacity to secrete biosurfactants or bioemulsifiers increasing the availability of hydrophobic compounds [65–67]. The cell walls of actinobacteria contain *ca.* 60% (dry mass) of the long-chained mycolate acids. These can be modified to form a thick and rigid envelope, which enables the microorganisms to decompose a wide range of hydrophobic compounds [67,68] and to enhance the uptake and bioavailability of phytosterols.

Hydrophobic phytosterols show in water a tendency to form agglomerates, which also limits their bioaccessibility. Addition of synthetic surfactants facilitated emulsification, inhibiting the aggregation of substrates, although the presence of artificial surfactants is usually toxic to bacteria and leads to necrosis and cell lysis [69]. It is known that in natural environments bacteria secrete bioemulsifiers or biosurfactants to make the hydrophobic substrates more available, for example mycolate-containing *Mycobacteriun* and *Rhodococcus*, which use phytosterols as carbon sources, synthesize biosurfactants [65]. Pursuing this property might become an alternative to the addition of artificial surfactants.

The mechanism of the cellular uptake of sterols, especially its transmembrane phase, is not yet fully understood, but its understanding would improve rational genetic modifications. Electron microscopy results led to a “flexible multi-component mesophase” model [63] providing a sharp concentration gradient in the mesophase between particles of sterols and the membrane. The model suggests that there should exist channels of proteins, stretching from the cytoplasm to the surface of the cell, capable of sterol binding or transformation, thus enhancing the transport. Further studies [51,70] confirmed the presence of a locus containing 10 genes, conserved in various mycolate-containing actinobacteria, which—if deactivated—resulted in inhibition of the sterol uptake.

The second major factor which limits conversion of phytosterols to steroid products is their toxicity to microbial cells. For example, AD and ADD are considered to inhibit cell growth and inhibit the enzyme activity in the sterol degradation pathway [71]. The tolerance of microorganisms towards toxicity of steroid products is one of the important features which can improve the strains to be able to transform phytosterols, although the mechanisms which allow the strains to stay resistant are still unknown. The improvement of the product yield is carried out by extracting the steroid products from the reaction media (*in situ* product recovery) and by creating mutants which are tolerant to the steroid products [62,71]. Amberlite XAD-7 resin, dimethyl siloxane and cyclodextrins are used to recover AD and ADD from the reaction media [72], also organic-aqueous two-phase systems are useful for the immediate recovery of the steroid product during the biotransformations [62].

The effective means of solving the problem of toxic products is to develop mutants with improved tolerance to toxicity of the steroids. This can be achieved by an increase in the efflux capability of the cell, or by directed evolutionary mutagenesis towards steroid-tolerant mutants. In the literature there are reports concerning improving the resistance and product yield by adding high amounts of androstanes to the culture after nitrosoguanidine mutagenesis, and further isolation of steroid-tolerant mutants [71].

In view of the fact that currently only the location of genes directly responsible for sterol catabolism is known in *Rhodococcus* and *Mycobacterium*, but usually not the function of the encoded proteins [51], the literature suggests that the immediate tasks are characterization of the mechanisms of phytosterol catabolism, phytosterol uptake, tolerance to toxic products, and global regulations involved in the sterol metabolism [46]. This might not be easy using known microorganisms of good performance, because the metabolic processes are complicated and interlaced. Therefore, instead of modifying microorganisms already known to transform phytosterols, the route suggested by Wang *et al.* is a complete reconstruction of the transformation pathway leading from phytosterols to the desired steroids in a heterologous host organism with suitable physiological trains. This can be achieved in two ways: First, by selecting a robust host which already is superior to the known phytosterol-transforming microorganisms with respect to phytosterol uptake and resistance to the product toxicity. This route would require thorough determination and understanding of the phytosterol metabolism, including degradation of the C-17 side chains. An alternative would be to follow the studies on yeast [73] where it is possible to achieve rerouting of the native biosynthesis of ergosterol to analogous brassicasterol and campesterol. Such reconstitution of the phytosterol transformation system in yeast has the advantage of being based on an already proven route and well-known microorganism and fermentation process, but one of the disadvantages is that yeast is vulnerable to the toxic effects of the steroid products; therefore, intensive research is necessary to utilize industrially this promising route.

A biocatalyst for industrial use should be easily cultured and non-pathogenic. This, unfortunately, is not the case with *Mycobacteria* which exhibit the overall best performance in transformations. There are however pathogens or opportunistic pathogens, not easily cultured, and slow in transformation (taking even a week). Future investigations might be therefore directed towards the less explored genera *Arthrobacter*, *Bacillus*, *Brevibacterium*, *Corynebacterium*, *Norcardia*, *Rhodococcus*, and others [46]. Experiments are ongoing on the isolation of new, useful biocatalysts from the natural environment, whose catalytic properties differ from currently known cases. An Actinomycete *Gordonia neofelifaecis*, isolated recently from the faeces of *Neofelis nebulosa*, carries out the conversion of cholesterol giving ADD as practically the sole product (*ca*. 90%) [53].

**Figure 13:**
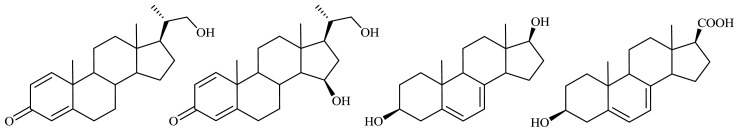
Products of microbiological degradation of sterols and their derivatives with proven biological activity.

Microbial sterol side chain degradation is also a powerful tool for generation of novel biologically active steroid compounds. Some products of this process and their derivatives are highly cytotoxic or possess immune system stimulating properties, effective against various infections (Figure 13). (20*S*)-20-Hydroxymethylpregna-1,4-dien-3-one showed strong cytotoxicity against HeLa cell lines; cytotoxic activity is also exhibited by its 15β-hydroxy derivative [74]. 3β-Hydroxyandrosta-5,7-diene-17-one—a product of microbial degradation of 3β-methoxy-methoxy-ergosterol, is a precursor of androsta-5,7-diene-3β,17β-diol [75]. Another steroidal 5,7-diene—3β-hydroxyandrosta-5,7-dien-17β-carboxylic acid—was tested in the context of inhibition of the proliferation of normal keratinocytes and normal and malignant melanocytes. It was also effective as a condition-dependent regulator of fibroblast proliferation, finally it stimulated leukemia cell differentiation [76].

### 2.2. Production of Optically Active β-Hydroxyacids

**Figure 14:**
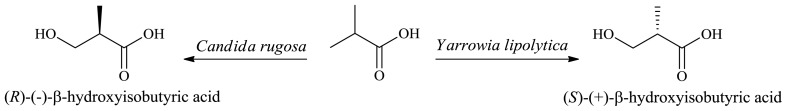
Enantioselective, microbiological hydroxylation of isobutyric acid.

Optically active β-hydroxyacids are the chiral building blocks in the asymmetric synthesis of drugs, vitamins, pheromones, fragrances and numerous other bioactive compounds [77]. Enantiomerically pure β-hydroxyacids can be obtained in various stereoselective enzymatic reactions: by reduction of the carbonyl group of β-ketoacids or β-ketoesters, oxidation of 1,3-diols, hydration of α,β-unsaturated acids [78], and by oxidation of an acid in the course of the partial β-oxidation cycle [77]. Significant practical applications among the products of the β-oxidation cycle were found for *R* and *S* enantiomers of β-hydroxyisobutyric acid (Figure 14), and l-carnitine.

**Figure 15:**
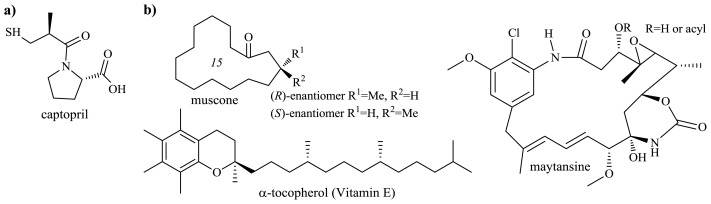
Structures of optically active products obtained from (**a**) (*R*)- and (**b**) (*S*)-β-hydroxyisobutyric acid intermediates.

Both enantiomers of β-hydroxyisobutyric acid find application in the synthesis of biologically active compounds, including drugs, vitamins and food additives [77,78] (Figure 15). Microbial hydroxylation of isobutyric acid is carried out with high enantioselectivity, although the molar yield of the product, particularly in the processes carried out at a higher concentration of substrate, was usually less than 50% [79,80], mainly due to degradation of the formed hydroxyacid by the producing strain. A high molar conversion yield was achieved using a mutant defective in β-hydroxyisobutyric acid dehydrogenase—the enzyme catalyzing subsequent to the hydration, reaction in the β-oxidation cycle [81]. For the industrial production of β-hydroxyisobutyric acid it is important to determine the optimal composition of the medium for cell growth and high activity of the enzymes catalyzing transformation, the elements necessary to ensure that the product is obtained with high molar conversion yield. Experiments have shown that both the cell growth of the microorganisms and the β-hydroxyisobutyric acid production decreased as the substrate concentration increased; a considerable degradation of the hydroxyacid product was observed when the substrate, isobutyric acid, was depleted in the medium. The production rate of β-hydroxyisobutyric acid increased as the glucose concentration decreased, while the conversion yield of isobutyric acid to β-hydroxyisobutyric acid showed an opposite trend. With controlled feeding of isobutyric acid and glucose, a high titer of β-hydroxyisobutyric acid was obtained by a fed-batch cultivation of the producing strain [82,83].

*Candida rugosa* IFO 0750 is the effective biocatalyst for the oxidation of isobutyric acid to (*R*)-β-hydroxyisobutyric acid [81,82]. Under optimal conditions the product was obtained with a concentration of 100 g/L (productivity 0.83 g/L/h), but the molar conversion yield of the product did not exceed 40% [82]. The low molar conversion yield was caused by the fact that the hydroxyacid was further metabolized into methylmalonic acid semialdehyde by (*R*)-β-hydroxyisobutyric acid dehydrogenase. The biocatalyst allowing higher yields of the product to be obtained was selected from among the mutants of *C. rugosa* IFO 0750 (mutations induced by UV/chemical treatments), which were not able to assimilate propionic acid. A stable mutant of *C. rugosa* MME 1259 transformed isobutyric acid to (*R*)-β-hydroxyisobutyric acid in a concentration of 150 g/L and with the molar conversion yield of over 80% [84]. (*R*)-β-Hydroxyisobutyric acid is a building block for the synthesis of captopril, which is known as a blood pressure-lowering agent [83] (Figure 15).

Strains of *Pseudomonas putida* and *Y. lipolytica* conduct enantioselective oxidation of isobutyric acid to (*S*)-β-hydroxyisobutyric acid [79,83] (Figure 14). A method of synthesis of (*S*)-β-hydroxyisobutyric acid from isobutyric or methacrylic acids by *Pseudomonas aeruginosa* was developed and patented [85]. (*S*)-β-Hydroxyisobutyric acid is an intermediate for the synthesis of α-tocoferol and calcimycin, both enantiomers of muscone, and maytansine—an antitumor-acting alkaloid [78,83] (Figure 15).

### 2.3. Production of l-Carnitine

**Figure 16:**
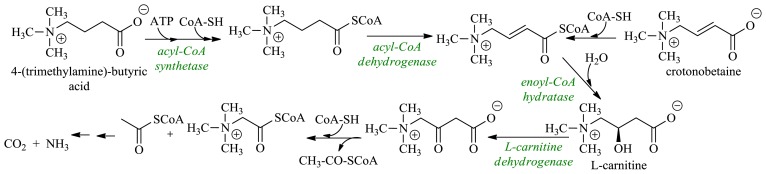
Scheme of microbiological degradation of 4-(trimethylamine)-butyric acid and crotonobetaine.

Among numerous important nutritional and medical applications found for l-carnitine [(*R*)-3-hydroxy-4-(trimethylamine)-butanoic acid] over the last 30 years, the most prominent are: protective action in heart diseases (*angina pectoris*) and asthma, weight-loss food supplementation, male infertility and osteoporosis treatment. Its metabolic role results from its participation in the β-oxidation cycle: carnitine helps transport fatty acids into the mitochondria, where the degradation process to acyl-CoA takes place. Global annual production of l-carnitine, reaching several thousand tons, is mostly carried out by microbial transformations, and successful artificial routes often include enzymatic kinetic resolution steps [86]. However, synthetic, racemic carnitine is not pharmacologically active; the (*S*) enantiomer inhibits carnitine acyltransferases and related proteins transporting carnitine through the mitochondrial membrane [87,88]. Microbial, enantioselective hydroxylation of 4-(trimethylamine)-butyric acid by a mutant HK13 is the core part of the synthesis of l-carnitine by the Swiss company Lonza [89]. The wild-type strain HK4, taxonomically related to *Agrobacterium* and *Rhizobium*, metabolizes the 4-(trimethylamine)-butyric acid and crotonobetaine to CO_2_ and ammonia (Figure 16), and the metabolite mixture contains identifiable, but small amounts of l-carnitine. The study indicates that l-carnitine, formed from the 4-(trimethylamine)-butyric acid (in the sequence of reactions: thioesterification with CoA → α,β-dehydrogenation → double bond hydration), is metabolized to CO_2_ and NH_3_. The HK4 strain was then subjected to mutagenesis and the mutant HK13 was isolated, which exhibited deficit of l-dehydrogenase. The mutant HK13 transforms the 4-(trimethylamine)-butyric acid into l-carnitine in quantitative yield.

A similar synthesis involving two enzymes (activation of crotonobetaine by thioesterification with CoA, and double bond hydration) was proposed for the transformation of crotonobetaine to l-carnitine in *Proteus* sp. [90]. This transformation, along with its analogue in *E. coli*[91], is, however, reversible and constitutes a pathway of carnitine degradation in microbes. Therefore, it is necessary to optimize the process in the desired direction: from crotonobetaine to l-carnitine. This was carried out by theoretical modeling of the kinetics of the process [92], as well as by various experimental means: increase in the permeability of the *E. coli* cells by addition of surfactants resulted in yields of up to 94% with the *E. coli* K38 T7-5KE32 strain [93]; increased availability of CoA and cofactor engineering resulting in redirection of metabolic fluxes were named as necessary steps in optimizing l-carnitine production by a variety of *E. coli* strains [94].

## 3. β-Oxidation in Synthesis of Achiral Products

### 3.1. Production of β-Hydroxy-β-Methylbutyric Acid

Numerous experimentations on animals have shown that β-hydroxy-β-methylbutyric acid exhibits anti-catabolic and anabolic effects and exerts positive impact on growth and health [95,96]. It is used as a dietary supplement for sportsmen, especially for bodybuilders, because it increases muscle fatigue resistance and promotes faster growth of muscle tissue [97]. In addition, β-hydroxy-β-methylbutyric acid induces proliferation of macrophages, stimulates phagocytosis [95], decreases muscle tissue reduction associated with autoimmune diseases (e.g., AIDS), prevents devastating effects of cancer, improves blood pressure and regulates the content of LDL-cholesterol [97].

**Figure 17:**
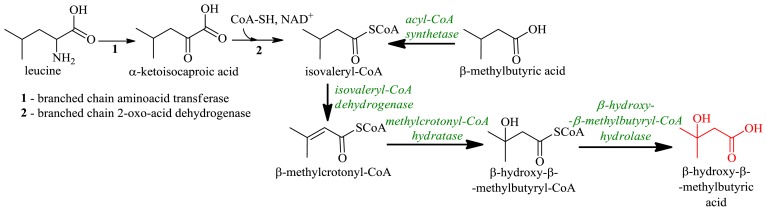
Microbial conversion of β-methylbutyric acid to β-hydroxy-β-methylbutyric acid is a part of the leucine catabolic pathway.

In mammals β-hydroxy-β-methylbutyrate is produced from leucine via transformation to α-ketocaproate (approximately 5% of leucine is metabolized into β-hydroxy-β-methylbutyrate) [97,98]. Enzymatic or microbiological approaches to β-hydroxy-β-methylbutyric acid syntheses are of interest due to the fact that the chemical method generates environmentally undesirable byproducts and residues. High levels of β-hydroxy-β-methylbutyric acid (0.38 g/L) can be synthesized by cultures of *Galactomyces reessii* (formerly *Endomyces reessii*) with β-methylbutyric acid (isovaleric acid) as the substrate [99,100], however the molar conversion yield of β-hydroxy-β-methylbutyric acid did not exceed 50% [99] (Figure 17).

Attempts have been made to verify the biocatalytic capabilities of *G. reessii* enzymes involved in β-methylbutyric acid to β-hydroxy-β-methylbutyric acid conversions. Activities of enzymes and the synthesis of β-hydroxy-β-methylbutyric acid from metabolic intermediates with cell-free extracts of *G. reessii* clearly showed that β-methylbutyric acid is transformed to β-hydroxy-β-methylbutyric acid via the leucine catabolic pathway [100] (Figure 17). The purified hydratase isolated from *G. reessii* catalyzed the hydration of crotonyl-CoA and methylcrotonyl-CoA; leucine and isovaleric acid are effective inducers of this enzyme. Competitive inhibition of *G. reessii* enoyl-CoA hydratase by acetyl-CoA, propionyl-CoA and acetoacetyl-CoA versus β-methylcrotonyl-CoA suggests a link between the hydratase and metabolites of the fatty acid β-oxidation [101]. An understanding of the kinetics, temperature, substrate specificities, inhibition and other aspects of the key enzyme could lead to further optimization of the whole cell process, and a considerable further improvement in β-hydroxy-β-methylbutyric acid yields.

### 3.2. Production of Vanillic Acid and Vanillin

Vanillic acid is one of the major components of “natural vanilla” aroma and it is used as a flavoring agent. It has also an antioxidant and antimicrobial activity and therefore it can be considered as a potential food preservative [102]. The highest amount of vanillic acid has been found in the roots of *Angelica sinensis*, a plant used in traditional Chinese medicine. Various studies have provided evidence of the pharmacological action of vanillic acid and its potential in the treatment of inflammation and immune disorders [103].

Vanillic acid is a valuable product for biotechnological applications since it is used as the starting material in the chemical synthesis of vanillin—one of the world’s most important flavoring compounds [104]. The high price of natural vanillin, with a demand significantly larger than the supply of vanilla extracted from vanilla beans, as well as the increase in consumer interest in products which can be labeled “natural”, are the combined causes of intensive research on the development of alternative technologies for the acquisition of vanillin. These efforts are mainly focused on the search of natural compounds that can be microbially converted to vanillin and the selection of an efficient biocatalyst capable of transforming the substrate. Since vanilin or vanillic acid are products in the microbial degradation of ferulic acid, eugenol and isoeugenol, these compounds are considered to be the best precursors for bioconversion processes [102,105]. The ferulic acid is an intermediate in the conversion of eugenol to vanillin by a number of microbial strains [106]. It is abundantly available from different natural sources, such as wood, sugar beet molasses, bran from corn and rice [107].

The bioconversion of ferulic acid to vanillin or vanillic acid has been studied with a wide range of microorganisms (see in [108]). A vanillin concentration not exceeding 16 g/L was obtained with *Streptomyces setonii*[109], and at least 7 g/L with *Amycolatopsis* sp. DSM 9992 [110]. The highest production of vanillin from an actinomycete using an adsorbent resin was 19.2 g/L with molar conversion yield of 54.4% [111]. Usually, the accumulation of vanillin in the culture of the microorganism is low, because it is further metabolized to vanillic acid by vanillin dehydrogenase (*vdh*) or other products utilized by microorganisms as a source of carbon and energy. The transformation of vanillin to vanillic acid or vanillic alcohol is a part of the strain’s defense mechanism—vanillin has toxic effects [104,112], and at concentrations above 1 g/L prevents growth of the vanillin-producing microorganisms. The increasing knowledge regarding enzymes that are responsible for the transformation of ferulic acid, as well as the identification of the genes coding them, offers opportunities of identifying metabolic pathways and construction of recombinant strains limiting further degradation.

**Figure 18:**
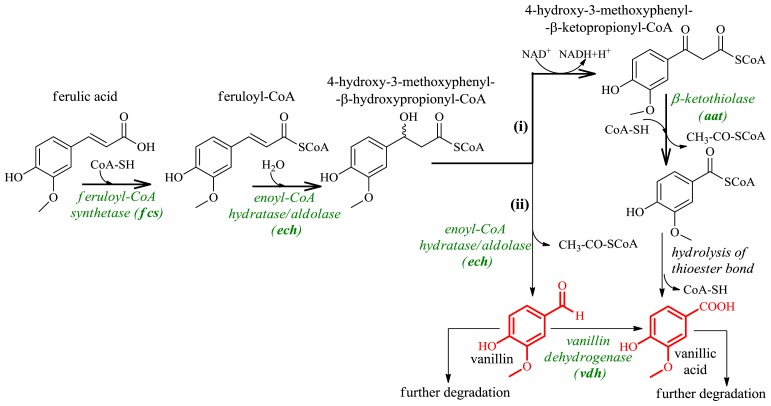
Enzymatic conversion of ferulic acid to vanillin and vanillic acid. Fine arrows indicate β-oxidative pathway. Thick arrows indicate non-β-oxidative degradation.

One of the pathways involved in the conversion of ferulic acid to vanillic acid or vanillin in microorganisms is the β-oxidation process that occurs by a mechanism analogous to the β-oxidation of fatty acids. Ferulic acid is activated to the CoA thioester by feruloyl-CoA synthetase (encoded by *fcs* genes), feruloyl-CoA is subsequently hydrated, and the pathway includes the thioclastic cleavage of 4-hydroxy-3-methoxyphenyl-β-ketopropionyl-CoA to acetyl-CoA and vanillyl-CoA, catalyzed by the β-ketothiolase (encoded by *aat* genes) (Figure 18, route (i)).

The β-Oxidative means of ferulic acid degradation was proposed with *Rhodotorula rubra*[113], *Rhodococcus* I24 [114], and white-rot fungus *Pycnoporus cinnabarinus*[115]. The penultimate strain tolerates high concentrations of eugenol, toxic to most microorganisms, and which therefore can be considered as a suitable candidate for vanillic acid or vanillin production from eugenol, a much cheaper substrate than the natural ferulic acid [112,116]. A technology for transforming ferulic acid, obtained from the waste residue of rice brain oil, into vanillin (with achieved yield of 2.8 g/L) was developed with the use of a combination of fungal strains *Aspergillus niger* and *Pycnoporus cinnabarinus*[117].

The much more common pathway of ferulic acid degradation in microorganisms is the mechanism in which, after hydration of feruloyl-CoA, 4-hydroxy-3-methoxyphenyl-β-hydroxypropionyl-CoA is cleaved to vanillin and acetyl-CoA (Figure 18, route (ii)). Both reactions are catalyzed by one enzyme enoyl-CoA hydratase/aldolase (encoded by *ech* genes). This mechanism has been described, among others, for *Pseudomonas* sp. HR199 [112], *P. fluorescens* BF13 [118], *Streptomyces setonii*[116], *S. sannanensis*[119], *Amycolatopsis* sp. HR167 [120], *Rhodococcus opacus* PD630 [114]. Recently, genetic engineering was applied to produce vanillin from ferulic acid using metabolically engineered *Escherichia coli*[105,106,121,122]. It was demonstrated that derivatives of the strain *P. fluorescens* BF13 accumulated vanillin if inactivation of the *vdh* gene was associated with expression of the *fcs* and *ech* genes [106]. This strain produced up to 8.41 mM of vanillin, which is the highest level for recombinant *Pseudomonas* strains.

## 4. Conclusions

There is a growing interest in exploiting microbial bioconversions for the production of high value-added products. In general, the advantages of biotechnological processes are: high substrate- or product specificity leading to only one isomer of the product, relatively mild reaction conditions and fewer environmental problems. Biotransformations involving β-oxidation reactions are exemplary for the practical utilization of enzymes and regulatory mechanisms developed in the course of the evolutionary refinement of cellular processes during the adaptation of microorganisms to the environmental conditions. Such biotransformations allow the production of valuable products from renewable, cheaper and natural raw materials; this includes also products unavailable via chemical syntheses. Various strategies have been pursued to achieve this goal. Finding the strains with the highest catalysis capability, modification of metabolic pathways to increase the accumulation of the desired product, as well as process optimization and recovery of the target products are desirable. However, until now, in most biotransformations the yields of the products are typically too low to make the biotechnological process workable; further studies are necessary to overcome the limitations found to date. Such studies aiming at novel efficient synthetic routes are stimulated by, and in turn also stimulate, progress in the fields of e.g., microbial physiology, biochemistry and genetics; thus, the respective metabolic pathways become clearer. This conclusion suggests that future studies will focus on the isolation of enzymes and genes involved in such pathways.

## Figures and Tables

**Table 1:** Some lactones with attractive fragrance properties for the food industry and perfumery available through biotechnology involving the β-oxidation cycle of fatty acids.

Fatty acid (substrate)	Lactone (product)	Odor description
ricinoleic	γ-decalactone	fatty, creamy, coconut, buttery, vanilla sweet
	3-hydroxy-γ-decalactone	odorless
	dec-3-en-4-olide	fruity, oily, fatty
	dec-2-en-4-olide	mushroom

jalapinolic or coriolic or linoleic	δ-decalactone	sweet, creamy, milky, peach, nut; peach, buttery on dilution

ipurolic	δ-octalactone	sweet, creamy, fatty with tropical and dairy nuances

linoleic	6-pentyl-2-pyrone	coconut, creamy, sweet, brown with a fatty waxy nuance

α-linolenic	δ-jasminlactone	powerful, creamy, jasmine-peachy-coconut

oleic	γ-dodecalactone	fatty, fruity, peach
